# Traumatic brain injury and alcohol intoxication: effects on injury patterns and short-term outcome

**DOI:** 10.1007/s00068-020-01381-6

**Published:** 2020-05-06

**Authors:** Henry Alexander Leijdesdorff, Juno Legué, Pieta Krijnen, Steven Rhemrev, Sanne Kleinveld, Inger Birgitta Schipper

**Affiliations:** 1 Department of Trauma Surgery, K06-R, Leiden University Medical Centre, PO Box 9600, NL 2300 RC Leiden, The Netherlands; 2 Haaglanden Medical Centre, Trauma Unit, The Hague, The Netherlands; 3 Department of Trauma Surgery, HAGA Hospital, The Hague, The Netherlands

**Keywords:** Alcohol intoxication, Blood alcohol concentration, Traumatic brain injury, Outcome, Mortality

## Abstract

**Purpose:**

A significant number of patients with traumatic brain injuries (TBI) are diagnosed with elevated blood alcohol concentration (BAC). Recent literature suggests a neuroprotective effect of alcohol on TBI, possibly associated with less morbidity and mortality. Our goal is to analyze the association of different levels of BAC with TBI characteristics and outcome.

**Methods:**

Adult patients with moderate to severe TBI (AIS ≥ 2) and measured BAC admitted to the Trauma Centre West (TCW), during the period 2010–2015, were retrospectively analyzed. Data included injury severity (AIS), length of hospitalization, admittance to the Intensive Care Unit (ICU) and in-hospital mortality. The association of BAC with ICU admittance and in-hospital mortality was analyzed using multivariable logistic regression analysis with correction for potentially confounding variables.

**Results:**

BACs were available in 2,686 patients of whom 42% had high, 26% moderate, 6% low and 26% had normal levels. Patients with high BAC’s were predominantly male, were younger, had lower ISS scores, lower AIS-head scores and less concomitant injuries compared to patients in the other BAC subgroups. High BACs were associated with a lower risk for in-hospital mortality (AOR 0.36, 95% CI 0.14–0.97). Also, patients with moderate and high BACs were less often admitted to the ICU (respectively, AOR 0.36, 95% CI 0.25–0.52 and AOR 0.40, 95% CI 0.29–0.57).

**Conclusion:**

The current study suggests that in patients with moderate to severe TBI, increasing BACs are associated with less severe TBI, less ICU admissions and a higher survival. Further research into the pathophysiological mechanism is necessary to help explain these findings.

## Introduction

Trauma remains the leading cause of severe morbidity and mortality around the world. Especially, traumatic brain injuries (TBI) contribute significantly to mortality and morbidity in trauma patients. The multidisciplinary nature of both the acute and long-term treatment of TBI patients is complex and expensive [1, 2]. TBI represents a major public health problem; its estimated incidence varies from 91 to 546 per 100,000 people per year in Europe and accounts for one-third of all trauma-related death in the United States [3, 4].

A strong correlation exists between alcohol intoxication and the increased risk for sustaining all types of injuries [5, 6]. TBI is no exception, with elevated blood alcohol concentrations (BACs) being demonstrated in 24–54% of the trauma patients diagnosed with TBI in Europe [4]. However, the influence of elevated BAC’s on the outcome of severely injured patients and the corresponding pathophysiological changes remain a controversial issue. Recent literature suggests a neuroprotective effect of alcohol on TBI [7–10], possibly resulting in lower mortality and morbidity rates compared to TBI patients without elevated BACs [11]. Despite this controversial suggestion and the questions that raises about cause-and-effects, only very few studies have addressed the influence of alcohol intoxication in general and of specific levels of BACs on TBI pattern and severity.

The aim of this study was to analyze the association between BACs, TBI pattern and patient outcome.

## Patients and methods

Data for this observational retrospective cohort study were obtained from the Dutch Trauma Centre West (DTCW) trauma registry. The DTCW trauma registry is part of the National Trauma Registry (LTR) and it encompasses the information of all trauma patients admitted to three level I trauma centers and nine level II and III trauma hospitals within the mid-Western part of the Netherlands, serving a population of 2.5 million. The DTCW trauma registry consists of Major Trauma Outcome Study (MTOS) [12] variables and pre-hospital data gathered from patients’ records by trained registrars. Trauma patients who only visited the emergency department without being admitted, as well as patients who deceased at the scene of the accident are not included in the registry. Also, in our study, patients who were transferred to hospitals not belonging to the DTCW region after initial presentation, were excluded from the analysis. The study was exempted from ethics review board approval, because the study made use of existing data sources and the patients had made no objection to use their coded data for scientific research.

An analysis of all adult (≥ 18 years) trauma patients who were admitted to one of the three regional level I trauma centers from January 1, 2010 to January 1, 2015 with moderate to severe TBI and measured BAC upon admission was performed. The analyzed data included age, gender, mechanism of injury and moderate to severe TBI (AIS ≥ 2) coded according to the Abbreviated Injury Scale 2005 update 2008 [13], injury severity coded according to the Injury Severity Score (ISS) [14], Glasgow Coma Scale (GCS) [15] on admittance, the Revised Trauma Score (RTS) [16]**,** and vital signs on admittance (including systolic blood pressure, heart rate, respiratory rate).

BACs in blood samples obtained in the ED on admission were gathered from patients’ laboratory records. These patients were then categorized in four groups: normal (< 0.1 g/L), low (0.1–1.0 g/L), moderate (1.0–2.3 g/L) and high (≥ 2.3 g/L).

The study outcomes were type and severity of TBI, length of hospitalization, admittance to and length of stay in the Intensive Care Unit (ICU) and in-hospital mortality.

### Statistical analysis

Group comparisons for continuous variables were performed using ANOVA for normally distributed data and using the Kruskal–Wallis test for skewed data. For comparing categorical variables, the Chi-square test was used. The association of BAC with ICU admittance and in-hospital mortality was analyzed using multivariable logistic regression analysis with correction for potentially confounding factors as categorical variables [age group, gender, head-AIS, associated severe injuries according AIS anatomical regions (AIS ≥ 3)]. GCS was not included in the multivariable analyses, because this parameter was missing in 20.9% of cases. All statistical analyses were repeated after exclusion of the patients with isolated brain concussions (AIS = 2), i.e., without other traumatic brain injuries. Data analysis was performed using IBM SPSS Statistics for Windows, version 23 (Armonk, NY: IBM Corp.). *p* values < 0.05 were considered as statistically significant.

## Results

### Demographic and clinical characteristics

During the studied period, 6061 patients with TBI were admitted to the level-1 trauma centers in the DTCW region. Of those, 216 were excluded because they were transferred between hospitals after their initial presentation and 51 were excluded due to missing or inconsistent data. BACs were reported in 2,686 of the remaining 5794 patients (44.3%).

Of these 2,686 patients, 1,120 (42%) had high BACs (≥ 2.3 g/L), 685 (26%) moderate BACs (1.0–2.3 g/L), 173 (6%) low BACs (0.1–1.0 g/L) and 708 (26%) had normal BACs (< 0.1 g/L). Patients with high BACs were predominantly male, were younger, had lower ISS scores and lower AIS-head scores compared to patients in the other BAC subgroups (Table 1). Differences in GCS between the BAC groups were more pronounced with GCS < 8 in 10.8% of patients with normal BAC versus 4.6% in the patients with the highest BAC (*p* < 0.0001).

**Table 1:** Comparison of demographics, injury characteristics and clinical outcome parameters of 2,686 hospitalized patients with traumatic brain injury and quantified blood alcohol concentration

Characteristics	Total (*n* = 2686)	High BAC (*n* = 1120)	Moderate BAC (*n* = 685)	Low BAC (*n* = 173)	Normal BAC (*n* = 708)	p*
Gender, *n* (%)						
Male	2002 (74.6)	903 (80.7)	514 (75.0)	123 (71.1)	462 (65.3)	< 0.0001
Age (years), mean (SD)	49.0 (18.7)	46.6 (14.9)	47.3 (19.6)	49.1 (20.1)	54.7 (21.4)	< 0.0001
Age by category, *n* (%)						
18–25	363 (13.5)	113 (10.1)	131 (19.1)	29 (16.8)	90 (12.7)	< 0.0001
26–55	1306 (48.6)	681 (60.8)	296 (43.2)	73 (42.2)	256 (36.2)	
56–75	781 (29.1)	303 (27.1)	206 (30.1)	53 (30.6)	219 (30.9)	
> 75	236 (8.8)	23 (2.1)	52 (7.6)	18 (10.4)	143 (20.2)	
Mechanism of injury, *n* (%)						
Traffic accident	929 (35.6)	290 (27.3)	231 (34.1)	53 (31.0)	355 (50.8)	< 0.0001
Low-energy fall	706 (27.0)	359 (33.7)	174 (25.7)	37 (21.6)	136 (19.5)	
High-energy fall	496 (19.0)	207 (19.5)	114 (16.8)	34 (19.9)	141 (20.2)	
Penetrating trauma	20 (0.8)	12 (1.1)	4 (0.6)	1 (0.6)	3 (0.4)	
Struck with blunt object	426 (16.3)	178 (16.7)	153 (22.6)	39 (22.8)	56 (8.0)	
Other	35 (1.3)	18 (1.7)	2 (0.3)	7 (4.1)	8 (1.1)	
ISS, median (range)	6 (4–75)	5 (4–50)	5 (4–59)	9 (4–75)	13 (4–59)	< 0.0001
By category, *n* (%)						
ISS < 16	2096 (78.0)	995 (88.8)	577 (84.2)	128 (74.0)	396 (55.9)	< 0.0001
ISS ≥ 16	590 (22.0)	125 (11.2)	108 (15.8)	45 (26.0)	312 (44.1)	
GCS, *n* (%)						
< 8	144 (6.8)	39 (4.6)	31 (5.8)	8 (5.9)	66 (10.8)	< 0.0001
8–12	142 (6.7)	60 (7.1)	22 (4.1)	7 (5.2)	53 (8.7)	
> 12	1839 (86.5)	751 (88.4)	478 (90.0)	120 (88.9)	490 (80.5)	
Head AIS, *n* (%)						
AIS = 2	2020 (75.2)	957 (85.4)	550 (80.3)	122 (70.5)	391 (55.2)	< 0.0001
AIS = 3	273 (10.2)	79 (7.1)	65 (9.5)	17 (9.8)	112 (15.8)	
AIS = 4	311 (11.6)	67 (6.0)	60 (8.8)	27 (15.6)	157 (22.2)	
AIS = 5	82 (3.1)	17 (1.5)	10 (1.5)	7 (4.0)	48 (6.8)	
Associated injuries AIS ≥ 3, *n* (%)	414 (15.4)	91 (8.1)	91 (13.3)	35 (20.2)	197 (27.8)	< 0.0001
Length of hospitalization in days, median (range)	2 (1–95)	2 (1–60)	2 (1–65)	2 (1–51)	4 (1–86)	< 0.0001
IC-admittance, *n* (%)	395 (15.1)	87 (8.0)	64 (9.6)	34 (19.9)	210 (30.9)	< 0.0001
Length of ICU stay in days, median (range)	2 (1–38)	2 (1–20)	2 (1–26)	2 (1–28)	3 (1–38)	< 0.0001
In-hospital mortality, *n* (%)	69 (2.6)	6 (0.5)	9 (1.3)	7 (4.1)	47 (6.7)	< 0.0001

*BAC* blood alcohol concentration (normal (< 0.1 g/L), low (0.1–1.0 g/L), moderate (1.0–2.3 g/L) and high (≥ 2.3 g/L));* SD* standard deviation;* ISS* Injury Severity Score;* GCS* Glasgow Coma Scale;* AIS* Abbreviated Injury Scale

* ANOVA for normally distributed continuous data, Kruskal-Wallis test for skewed continuous data and Chi-square test for categorical data.

### TBI pattern

The most frequently diagnosed TBI in the study group was a cerebral concussion (76.5%), followed by injuries to the cerebrum (22.2%) (Table 2). The majority of patients were diagnosed with an isolated cerebral concussion (69.2%). Patients with elevated BACs had significantly less injuries to the cerebrum, skeletal injuries and cerebral concussions (*p* < 0.0001). Also, a trend was observed between patients with different BACs with progressively more concussions and progressively less cerebral and skeletal injuries in patients with increasing BACs (Table 2).

**Table 2:** Anatomical location and type of traumatic brain injury in hospital-admitted trauma patients with available quantified blood alcohol concentrations

	Total (*n* = 2686)	High BAC (*n* = 1120)	Moderate BAC (*n* = 685)	Low BAC (*n* = 173)	Normal BAC (n = 708)	*p*
Brain stem	3 (0.1)	1 (0.1)	0 (0)	0(0)	2 (0.3)	0.41
Cerebellum	60 (2.2)	24 (2.1)	12 (1.8)	4 (2.3)	20 (2.8)	0.59
Cerebrum	597 (22.2)	139 (12.4)	118 (17.2)	48 (27.7)	292 (41.2)	< 0.0001
Contusion	286 (10.6)	68 (6.1)	61 (8.9)	22 (12.7)	135 (19.1)	< 0.0001
Hematoma epi- or extradural	75 (2.8)	12 (1.1)	10 (1.5)	10 (5.8)	43 (6.1)	< 0.0001
Hematoma intracerebral	47 (1.7)	6 (0.5)	11 (1.6)	4 (2.3)	26 (3.7)	< 0.0001
Hematoma subdural	267 (9.9)	63 (5.6)	47 (6.9)	24 (13.9)	133 (18.8)	< 0.0001
Subarachnoid hemorrhage	239 (8.9)	60 (5.4)	44 (6.4)	16 (9.2)	119 (16.8)	< 0.0001
Skeletal	313 (11.7)	77 (6.9)	65 (9.5)	17 (9.8)	154 (21.8)	< 0.0001
Base fracture	80 (3.0)	13 (1.2)	19 (2.8)	7 (4.0)	41 (5.8)	< 0.0001
Vault fracture	151 (5.6)	43 (3.8)	28 (4.1)	6 (3.5)	74 (10.5)	< 0.0001
Close vault fracture	58 (2.2)	10 (0.9)	13 (1.9)	3 (1.7)	32 (4.5)	< 0.0001
Cerebral concussion	2055 (76.5)	960 (85.7)	567 (82.8)	124 (71.7)	404 (57.1)	< 0.0001

Results are presented as numbers (%) of patients with that specific injury. Patients may have multiple traumatic brain injury locations or types

*BAC* blood alcohol concentration (normal (< 0.1 g/L), low (0.1–1.0 g/L), moderate (1.0–2.3 g/L) and high (≥ 2.3 g/L))

### Associated injuries

Patients with TBI and elevated BACs had significantly less associated severe injuries (AIS ≥ 3) in anatomical regions other than to the head compared to patients with normal BACs (Table 1). Also, the percentage of patients with associated severe injuries decreased significantly with increasing BACs from 20.2% in the low BAC group to 8.1% in the high BAC group (Table 1).

**Fig. 1:**
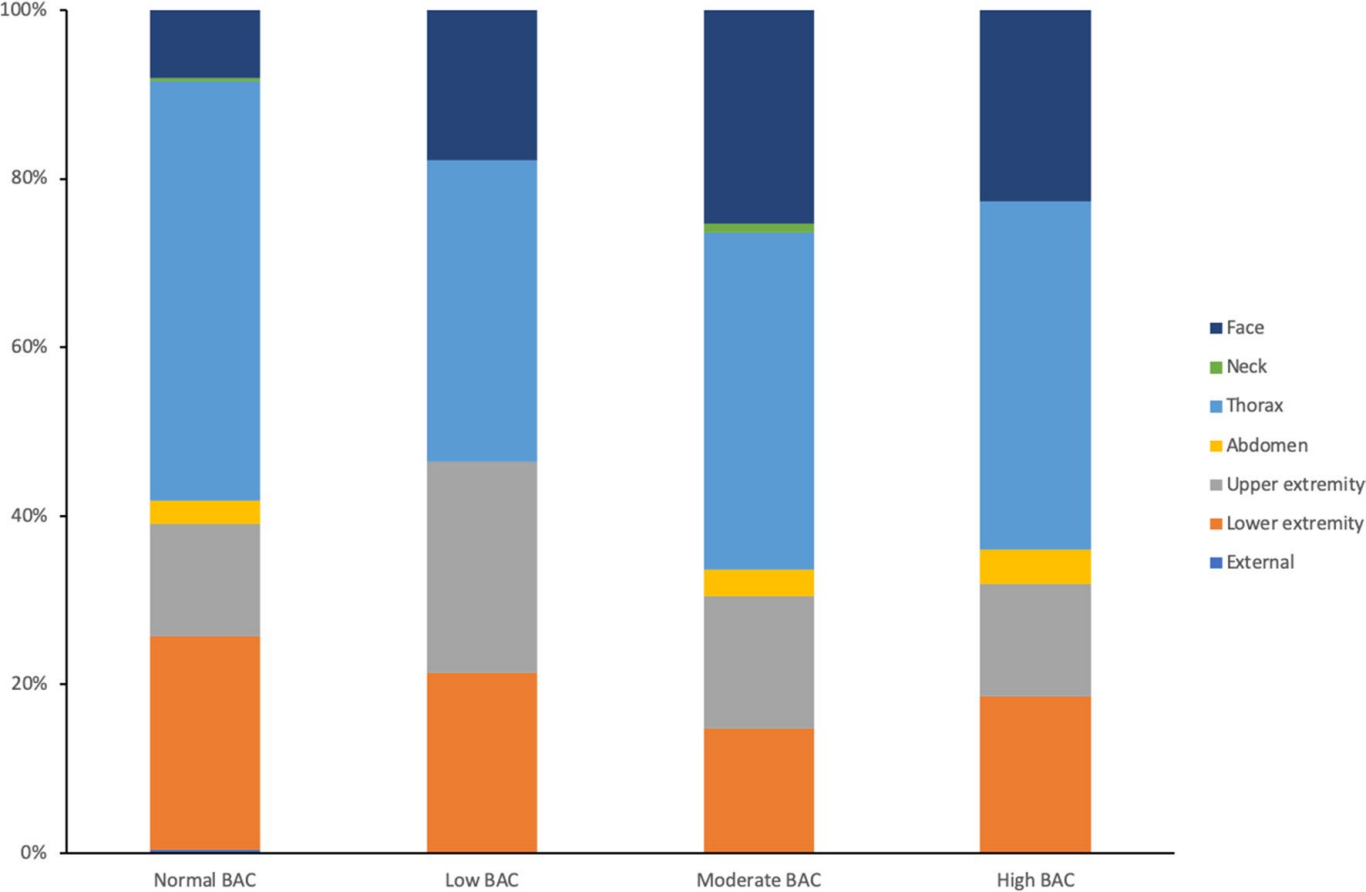
Distribution of associated injuries per anatomical region (according to the AIS classification) in alcohol-intoxicated and non-alcohol intoxicated trauma patients with traumatic brain injury. The External category comprises of burns

The most frequently diagnosed severe associated injuries in all groups were thoracic injuries (lung contusions and rib fractures). Second most common were injuries to the lower extremities (mainly femur fractures) in patients in the normal BAC group, and injuries to the face (predominantly injuries to the orbita) in patients with moderate and high BAC’s (Fig. 1).

### Clinical outcome

The median length of hospitalization in the total group was 2 days (range 1–95) and was highest in the group with normal BACs (median 4 days, range 1–86; Table 1). Also, ICU admittance and ICU length of stay were highest in the patients with normal BACs (Table 1). When adjusted for age, gender, AIS head and the presence of associated severe injuries, the risk of ICU admittance for patients with moderate and high BACs was significantly lower compared to patients with normal BAC’s (respectively, AOR 0.36, 95% CI 0.25–0.52 and AOR 0.40, 95% CI 0.29–0.57) (Table 3).

**Table 3:** Predictive value of BACs for in-hospital mortality and ICU admittance in patients with traumatic brain injury

	Risk of in-hospital mortality^a^		Risk of ICU admittance^b^	
	Crude OR (95% CI)	Adjusted OR^c^ (95% CI)	Crude OR (95% CI)	Adjusted OR^c^ (95% CI)
High BAC	0.08 (0.03–0.18)	0.36 (0.14–0.97)	0.19 (0.15–0.25)	0.40 (0.29–0.57)
Moderate BAC	0.19 (0.09–0.38)	0.60 (0.26–1.38)	0.24 (0.17–0.32)	0.36 (0.25–0.52)
Low BAC	0.60 (0.26–1.34)	1.39 (0.54–3.57)	0.56 (0.37–0.84)	0.72 (0.43–1.19)
Normal BAC	Ref.	Ref.	Ref.	Ref.

*BAC* blood alcohol concentration (normal (< 0.1 g/L), low (0.1–1.0 g/L), moderate (1.0–2.3 g/L) and high (≥ 2.3 g/L)); *ICU* intensive care unit; *OR* odds ratio; CI, confidence interval

^a^Results are based on 2672/2686 (99.5%) of patients with available BAC

^b^Results are based on 2608/2686 (97.1%) of patients with available BAC

^c^Adjusted for age group, gender, AIS head and presence of associated severe injury (AIS ≥ 3)

In-hospital mortality was highest in patients with normal BACs (Table 1). Although the association between alcohol intoxication and in-hospital mortality was less strong after adjustment for confounding, the adjusted risk of death after admission to the hospital remained lower in patients with high levels of BAC (AOR 0.36, 95% CI 0.14–0.97) (Table 3).

### Outcome in the TBI patients without isolated concussion

All statistical analyses were performed again to determine if the results were comparable when the patients with isolated cerebral concussions were excluded. After exclusion, 827 patients (30.8%) without isolated concussions and with reported BACs were analysed; 368 patients with normal BACs, 61 with low BACs, 174 with moderate BACs and 224 with high BACs. Clinical and demographical characteristics were comparable to the results for the total group described above. The estimated association of different levels of BACs with in-hospital mortality was somewhat lower than those in the total group and not statistically significant for all BAC levels (for high BAC: AOR 0.46, 95% CI 0.17–1.26). Also, patients with moderate or high BACs without isolated concussion were less likely to be admitted to the ICU (for moderate BAC: AOR 0.41, 95% CI 0.26–0.64; for high BAC: AOR 0.48, 95% CI 0.31–0.74).

## Discussion

The aim of the study was to examine the controversial topic of alcohol intoxication at the time of injury and its assumed protective effect on the short-term outcome in TBI patients. Also, the TBI pattern was examined in relation to different BACs at the time of injury.

The findings of this study are in line with previous studies reporting possible protective effects of alcohol consumption in relation to TBI**:** Patients with high BACs were predominantly male, were younger, had lower ISS scores and lower AIS-head scores compared to patients in the other BAC subgroups of TBI patients. Increasing BACs were associated with less severe TBI, less concomitant injuries, less ICU admissions and a higher survival.

### TBI pattern

Cerebral injuries (contusions, subdural hematomas and subarachnoid hemorrhage) and concussions were the most frequently diagnosed TBIs in our study population. However, with rising BACs, these were diagnosed less often. These findings are in accordance with the results of the previous studies. Talving et al. [17] found that in patients with isolated severe TBI, blood alcohol levels were not associated with overall head injury severity. Lustenberg et al. [18] showed that in their study population subarachnoid, intraparenchymal and subdural hematoma were the most frequent injuries, followed by skull fractures. Their group with alcohol-intoxicated patients showed significantly less skull fractures than the non-intoxicated patients, which was also the case in our study. Again, another study pointed out that acute alcohol intoxication was not associated with type and number of diffuse axonal injury lesions and intraventricular bleedings [19].

### Effect on mortality

The occurrence of associated injuries has been found to be associated with higher mortality and longer hospital and ICU stay in patients with TBI [20]. Our study showed a significantly lower risk for in-hospital mortality in all intoxicated patients and especially in patients with high levels of BAC. Berry et al. classified BACs according to the same categories as used in our study [9]. Despite the fact that they did not include patients with moderate TBI (AIS = 2) nor those with associated severe injuries (AIS ≥ 3), they also found that high levels of BAC were independently associated with an improved survival. Talvin et al. published similar effects on survival [17]. Several other studies compared intoxicated with non-intoxicated patients and also found lower risks of mortality in intoxicated patient groups [21–23]. However, some other studies did not find these effects of alcohol on mortality [24–26]. For example, Chen et al. concluded that the possible protective effect of alcohol no longer existed after correction for residual confounding variables such as causes and intents of TBI and injury severity scores (ISS). The adjusted in-hospital mortality even appeared to increase [27]. It should be noted again that all previous studies only evaluated patients with severe TBI (AIShead ≥ 3) and are not completely comparable with our study. A recently conducted meta-analysis evaluated mortality in relation to TBI and alcohol intoxication in 15 studies, including the previously mentioned studies. It showed no significant difference in mortality between alcohol-intoxicated and non-intoxicated patients nor between low levels and high levels of BAC [28]. Unfortunately, due to the small sample sizes of some studies and only few of the included studies examined comparable outcomes, the reliability of this meta-analysis is limited [20].

### Effect on length of hospital stay, ICU admittance and ICU length of stay

Rising levels of BAC were associated with a shorter length of hospital stay when compared to the group with normal BACs. This finding is in line with Berry et al. [9] who described a statistically significant difference in length of hospital stay for different alcohol concentration categories; patients with high BACs had less hospitalization days than patients with normal BACs. Nevertheless, other studies, as well as the recently published meta-analysis, showed no differences in length of hospital stay [17, 22, 23, 26, 28].

The shorter time at the ICU of patients with moderate and high BACs may be explained by the fact that especially in patients with high BACs, the alcohol intoxication affects the initial level of consciousness. In cases where alcohol intoxication is the cause of decreased consciousness, this will normalize over limited time as the BAC decreases. When patients have recovered consciousness, ICU admittance will no longer be necessary. In the literature, only one study showed a significant difference between the different BACs, with a trend towards less ICU admittances and shorter length of stay in the high BAC group [9]. The recently conducted meta-analysis included studies with patients aged > 16 and without penetrating TBI, and showed a shorter length of ICU stay in intoxicated patients [28]. This was mainly due to the study conducted by Salim et al. [22] that had a large study size and found that ICU length of stay was shorter in the intoxicated group.

### Explanation of the effects found

Different potential mechanisms have been proposed to account for the protective effects of alcohol on TBI. A recent study showed that alcohol intoxication may have protective effects in TBI at behavioral and histological level. It suggests that when alcohol intoxication is present at the moment right before trauma, it significantly decreases the trauma-induced transcriptional responses of hippocampal neurons [29]. Another possible mechanism is the inhibition of N-Methyl-D-aspartic acid receptors (NMDAr). NMDAr is associated with neuronal cell death due to a chain reaction that occurs when NMDAr overactivation leads to a major release of excitatory neurotransmitters [11]. Alcohol acts as a NMDAr antagonist and inhibits this process. Another popular theory is that alcohol blunts the adrenergic response that occurs when a person sustains a TBI [10, 11, 30–32]. Obviously, the exact mechanism by which alcohol may enhance survival is not yet fully understood at this time. Further studies that provide further insight in this mechanism may also be of use in the development of therapeutic agents for the treatment of TBI.

## Limitations

The limitations that result from a retrospective research set-up all do apply. Especially, the fact that we obtained 2686 BAC serum levels from 5794 TBI patients has potentially introduced a bias. We did, however, also analyze the group of TBI patients of which we did not have BACs (data not presented) and found that alcohol intoxication is associated with less severe TBI, shorter length of hospitalization and ICU admission, and higher survival.

Every effort was made to code injuries accurately in our trauma registry, but because of the retrospective design of the study, a possible selection bias may have been introduced concerning the interpretation of injury patterns and injury severity, therefore influencing the AIS and ISS coding. Also, it was impossible to retrieve all the missing GCS data because of the retrospective design of our study. Thus, it can be assumed that some of our findings are the result of residual confounding.

Another issue to consider is the fact that we did not distinguish between acute alcohol intoxication and chronic alcohol consumption, nor was history of alcohol use or abuse documented. Trauma patients with behavioral and biochemical evidence of chronic alcohol abuse have been associated with higher complication rates, worse outcomes and longer length of stay compared to acutely intoxicated patients [33]. It is plausible that patients with chronic alcohol abuse and TBI also have a higher complication rate, worse outcomes and longer length of stay when compared to acutely intoxicated patients as has been described with other injuries. Their presence in the current study would attenuate the clear effects found and exclusion of these patients probably would magnify these effects.

## Conclusions

The current study results suggest that increasing levels of BAC in patients with moderate and severe TBI are independently, i.e. after correcting for confounding variables, associated with lower injury severity, shorter length of hospital and ICU stay and improved survival. Controversy remains and further clinical and basic research is necessary into the pathophysiological mechanisms to help explain the outcomes found.
